# Prevalence and Unmet Need for Mental Healthcare of Major Depressive Disorder in Community-Dwelling Chinese People Living With Vision Disability

**DOI:** 10.3389/fpubh.2022.900425

**Published:** 2022-06-22

**Authors:** Bao-Liang Zhong, Yan-Min Xu, Yi Li

**Affiliations:** ^1^Department of Psychiatry, Wuhan Mental Health Center, Wuhan, China; ^2^Department of Clinical Psychology, Wuhan Hospital for Psychotherapy, Wuhan, China

**Keywords:** major depression, vision disability, prevalence, correlate, mental health services

## Abstract

**Objective:**

Mental health services have been recognized as an essential part of the comprehensive eye care services, but data regarding the mental health of people living with vision disability (PLwVD) remain very limited. This study examined the epidemiology of major depressive disorder (MDD) among Chinese PLwVD, as well as their perceived needs for and utilization of mental health services.

**Methods:**

By using stratified cluster sampling method, a total of 1,753 PLwVD were successfully recruited from 73 urban communities and 169 rural villages in Wuhan, China, and interviewed with the Mini-international Neuropsychiatric Interview 5.0. Standardized questions were used to measure perceived mental healthcare needs and use of mental health services of PLwVD with MDD.

**Results:**

The one-month prevalence of MDD was 24.4%. Among the PLwVD with MDD, 26.0% perceived needs for mental healthcare and only 1.2% sought treatment from mental health specialists for their emotional problems. Factors associated with MDD were middle age [vs. young adulthood, odds ratio (OR) = 1.96, *P* < 0.001], older adulthood (vs. young adulthood, OR = 1.79, P = 0.004), being never-married (vs. married, remarried, and cohabiting, OR = 1.96, *P* < 0.001), being separated, divorced, and widowed (vs. married, remarried, and cohabiting, OR = 12.30, *P* < 0.001), a low level of objective social support (vs. high, OR = 1.83, *P* < 0.001), currently drinking (OR = 1.81, *P* < 0.001), having childhood-onset eye conditions (OR = 1.89, P = 0.005), and having difficulties in performing daily activities (OR = 2.78, *P* < 0.001).

**Conclusions:**

Chinese PLwVD are at high risk for MDD and have a high level of unmet need for mental healthcare. Public strategies are warranted to improve the mental health literacy of PLwVD and make the mental health services available, accessible, and affordable for PLwVD.

## Introduction

Due to population growth, aging, urbanization, and lifestyle changes, there will be a substantial increase in the global estimate of number of people with eye conditions, vision impairments, and vision disability in the coming decades (1). According to the World Report on Vision by World Health Organization (WHO), the current number of people living with a vision impairment has been at least 2.2 billion in the world (1). Global needs associated with eye conditions and vision impairments are projected to increase dramatically in coming years and there have been increasing calls for comprehensive and integrated people-centered eye care services (2–4). Mental health services have been recognized as an essential part of the comprehensive eye care services but mental health is often underestimated and under prioritized by eye care workers and persons with eye conditions (5). Accurate assessment of the met and unmet mental health needs of persons with eye conditions is important to ensure effective mental healthcare planning and high-quality eye care.

Eye conditions and vision impairments are associated with many negative consequences such as vision loss, psychological distress, impaired everyday functioning, social isolation and loneliness, uncertainty in illness, and deteriorated socioeconomic status; therefore, compared to normally-sighted persons, persons with eye conditions and vision impairments are more likely to develop mental health problems, in particular depression (1, 6–10). For example, the prevalence rates of depressive symptoms in middle-aged and older adults with glaucoma, patients with cataracts, patients attending eye clinics, patients with chronic thyroid eye disease, patients with legal blindness, attendees at low-vision rehabilitation centers, and patients with dry eye disease are 10.9, 23.9, 25.0, 28.0, 29.0, 43.0, and 61.0%, respectively (11–17).

In general, instruments for assessing depression include self-report and diagnostic interview measures (18). The former defines the presence of depression based on cut-off scores of scales of depressive symptoms such as 9-item Patient Health Questionnaire and Hospital Anxiety and Depression Scale. These scales, while they are cost-effective and time-efficient, are only screeners to identify individuals with depressive symptoms (19). By contrast, the diagnostic interviews define the presence of depression based on both depressive symptoms and their associated clinically significant distress or impairment in social, occupational, or other important domains of functioning (20). Therefore, diagnostic interview-based assessment findings are more clinically relevant than self-report-based assessment findings. Because nearly all existing studies assessed depression in persons with eye conditions and vision impairments by using self-report scales (11–17), one significant limitation of prior studies is the limited clinical significance of their assessments of depression. In addition, the other significant limitation of previous studies is the potential selection bias in samples of persons with eye conditions and impairments, since samples of most available studies were recruited from clinical settings (6, 12–14, 16, 17).

While some eye conditions result in impairment in one or more vision functions, many eye conditions do not cause vision impairments (1). Unlike the medical nature of vision impairment, vision disability is constructed socially, culturally, and in public policy, which occurs when a person with eye condition experiencing vision impairments or blindness and facing physical, social, and attitudinal environmental barriers such as no access to assistive products and rehabilitation services and no barrier-free facilities in public transportations (21). Because people living with vision disability (PLwVD) have been subject to social, financial, physical and attitudinal barriers, PLwVD represent the more vulnerable subgroup of the people with eye conditions and vision impairments. Accordingly, studies focusing on the mental health of PLwVD carry the implication that the physical and social environment must be adjusted to improve their social participation on an equal basis with others.

Unfortunately, empirical data on the mental health of PLwVD are very limited. As far as we know, only a study by Li and colleagues examined depressive symptoms in Chinese PLwVD in 2011 (22). This study found that as high as 40.4% of the PLwVD had depressive symptoms, as measured by using the Center for Epidemiological Studies Depression Scale. However, because of no rigorous psychiatric interviews and no assessment of mental health needs, the proportions of PLwVD whose depressive symptoms are severe enough to meet the clinical diagnostic criteria of major depressive disorder (MDD) and PLwVD with MDD who received treatment from mental health specialists remain unknown.

In China, 1.29% of the general population, 18.2 million, are PLwVD (23). During the past decades, nationwide efforts have been implemented to improve the quality of life and social participation of PLwVD in China, including public disability subsidies, rehabilitation services, inclusive education, human rights protection, and barrier-free environment (24). However, mental health services have long been neglected in the eye rehabilitation services and disability research in China (25). To facilitate the planning and provision of mental health services, this study investigated the prevalence and correlates of MDD among Chinese community-dwelling PLwVD, as well as perceived need for and utilization of mental health services of PLwVD with MDD. Because vision disability is the results of the interaction between people living with vision impairments and barriers in the physical and social environment, we hypothesized that MDD would be prevalent in PLwVD, and it would be associated with some socio-demographic and clinical characteristics such as social support and the severity of vision impairments.

## Materials and Methods

### Sampling and Participants

The China Disabled Persons' Federation (CDPF) is a nationwide semi-government organization of people with disabilities (PwD) and its mission is to promote PwD's full social participation on an equal basis with others, protect PwD's legitimate rights, and provide government financial assistance and other public services to PwD (22). In China, the official definition of vision disability is “a significant loss of vision or a significant visual field defect in both eyes that cannot be corrected and the vision impairment is so extensive that it impairs an individual's daily life and social participation”(26). Therefore, not all Chinese persons with eye conditions or vision impairments are PLwVD; rather only those whose vision disability is strictly evaluated and ascertained according to the standard procedures at officially designated hospitals can be PLwVD (22, 24). These ascertained persons are referred to register with the CDPF and awarded disability certificates, which contain holders' demographic information and type and degree of disability and qualify holders for government financial supports (22).

This cross-sectional survey was carried out in Wuhan, China, between April and August, 2019. Wuhan is the largest city in central China with 11.2 million residents in 2019 (27), and, at the time of this survey, there were over 41 thousand PLwVD registered in the CDPF system of Wuhan, China. Because community-based household sampling is not feasible, PLwVD were recruited from the CDPF registration system by using a multi-stage stratified cluster sampling approach. In the first stage, two of the seven urban districts and two of the eight rural districts were randomly selected from this city. The second stage adopted probability proportionate to size method to randomly selected 73 of the 379 urban communities and 169 of the 874 rural villages from the four selected districts. In the final stage, a total of 2,374 PLwVD residing in the selected communities and villages were invited to participate in this study. Among these identified participants, 1,753 (73.8%) agreed to participate in and completed the survey. Eligible participants were registered PLwVD, aged 16 years old and above, and residents of selected communities and villages of the four districts in Wuhan.

We used the Power Analysis & Sample Size 11.0.7 (NCSS, LLC. Kaysville, Utah, USA) to calculate the sample size for this prevalence study. A minimum required sample size of 1,982 was generated according to the following parameters: a 20% prevalence of MDD based on the pilot survey, a two-sided 0.05 type I error rate, a confidence interval width of 0.04, and an 80% survey response rate.

The survey proposal was approved by the Institutional Review Board of Wuhan Mental Health Center. All participants and their guardians (if necessary) provided written informed consent before the interview.

### Procedures and Instruments

Face-to-face and in-person household interviews were performed to collect data. The validated Chinese Mini-international Neuropsychiatric Interview 5.0 (MINI 5.0) was used to assess the presence of MDD according to the Diagnostic and Statistical Manual of Mental Disorders, Fourth Edition, Text Revision (DSM-IV-TR) (28). In China, the MINI 5.0 has been widely used in community-based epidemiological surveys of mental disorders (24, 29–32). Interviewers of this study were fifteen psychiatrists who had at least three-year clinical experiences and had been trained to administer the MINI 5.0. Before the formal survey, these interviewers attended an inter-rater reliability test that involved eight patients with mood disorders, six patients with anxiety disorders, two patients with schizophrenia, and three healthy volunteers. The kappa value between interviewers for mental disorders was 0.81. When necessary, family caregivers of PLwVD were additionally interviewed to clarify their responses. In this study, the timeframe for MDD was the past month before the interview.

The perceived need for mental healthcare and utilization of mental health services of participants diagnosed with MDD were assessed by asking: “Do you recognize that you need treatment from mental health specialists because of your recent emotional problems? These specialists include psychiatrists, psycho-therapists, and psychological counselors” and “Did you receive any treatment from mental health specialists for your recent emotional problems?” respectively (24, 33).

Social support was assessed with the validated Chinese Social Support Rating Scale (SSRS), which has ten items and evaluates three domains of social support: objective, subjective, and utilization of social support (34). The higher total scores denote higher level of social support. In this study, each domain score was split at the median to form groups of high vs. low levels of social support.

Demographic variables in the survey questionnaire were age, sex, educational attainment, marital status, employment status, self-rated family economic status (poor, fair, good), residence place, having disability allowance (yes, no), and living arrangement (with family members, with others, alone). Age was divided into three categories: young adulthood (16–35 years), middle age (36–55 years), and older adulthood (56 years and older) (35). In this study, marital status was classified into three categories: married, remarried and cohabiting, never-married, and separated, divorced and widowed.

Current smokers and drinkers were those who were currently smoking at least one cigarette per day and had smoked for at least half a year and drank alcoholic beverage at least once per month in the past 6 months, respectively (36–39).

Clinical factors included age at onset of the eye condition (at birth, childhood, adulthood), severity of vision impairment, and ability to perform normal daily activities. In China, the assessment of severity of vision impairment is based on visual acuity and field in the better eye of an individual, which has two levels: blindness and low vision (40). The validated Chinese Activity of Daily Living Scale (ADLS) was used to assess PLwVD's ability to perform normal daily activities required to meet self-care needs and maintain independent households (41). The ADLS has 14 items and its total score ranges between 14 and 56. In China, a cutoff score of 21 or more suggests a dependency on others to perform activities of daily living (41).

### Statistical Analysis

SPSS software version 20.0 was used to analyze the data. Prevalence rates of MDD, perceived mental health needs, and utilization of mental health services were calculated. Chi-square test was used to compare rates of MDD between/across subgroups according to demographic, lifestyle, and clinical characteristics. Multiple binary logistic regression with a backward stepwise entry of all statistically significant factors in the Chi-square test was adopted to identify factors associated with MDD. We used odds ratios (ORs) and their 95% confidence intervals (95% CIs) to quantify associations between factors and MDD. The statistically significant level was set at two-sided *P* ≤ 0.05.

## Results

### Sample Characteristics

Among the 1,753 completers, 1,073 (61.2%) were men and the mean age was 48.5 years (standard deviation: 15.7, range: 16–93). In total, 979 (55.8%) were blind and 885 (50.5%) were dependent on others to perform daily activities. Other detailed demographic, lifestyle, and clinical characteristics are displayed in Table 1.

**Table 1:** Characteristics of participants and prevalence rates of major depressive disorder by demographic, lifestyle, and clinical factors.

**Variable**		***n* (%)**	**Number of adults with major depression (%)**	**χ^2^**	***P***
Sex	Males	1,073 (61.2)	289 (26.9)	9.959	0.002
	Females	680 (38.8)	138 (20.3)		
Age-group (years)	16–35	382 (21.8)	69 (18.1)	10.588	0.005
	36–55	912 (52.0)	236 (25.9)		
	56+	459 (26.2)	122 (26.6)		
Education	Primary school and below	504 (28.8)	164 (32.5)	30.853	<0.001
	Middle school	1,090 (32.2)	241 (22.1)		
	College and above	159 (9.1)	22 (13.8)		
Marital status	Married, remarried, and cohabiting	1,444 (82.4)	327 (22.6)	37.444	<0.001
	Never-married	292 (16.7)	86 (29.5)		
	Separated, divorced, and widowed	17 (1.0)	14 (82.4)		
Employment status	Yes	766 (43.7)	117 (15.3)	60.932	<0.001
	No	987 (56.3)	310 (31.4)		
Self-rated family economic status^*^	Fair and good	330 (18.8)	65 (19.7)	4.794	0.029
	Poor	1,423 (81.2)	362 (25.4)		
Having disability allowance	Yes	836 (47.7)	198 (23.7)	0.394	0.530
	No	917 (52.3)	229 (25.0)		
Residence place	Urban	910 (51.9)	163 (17.9)	42.677	<0.001
	Rural	843 (48.1)	264 (31.3)		
Living arrangement	With family members	1,412 (80.5)	333 (23.6)	2.364	0.124
	With others or alone	341 (19.5)	94 (27.6)		
Objective social support^**^	≥6	1,393 (79.5)	312 (22.4)	14.151	<0.001
	<6	360 (20.5)	115 (31.9)		
Subjective social support^**^	≥18	1,009 (57.6)	234 (23.2)	1.757	0.185
	<18	744 (42.4)	193 (25.9)		
Utilization of social support^**^	≥7	1,055 (68.2)	222 (21.0)	15.809	<0.001
	<7	698 (39.8)	205 (29.4)		
Currently smoking	No	1,215 (69.3)	291 (24.0)	0.357	0.550
	Yes	538 (30.7)	136 (25.3)		
Currently drinking	No	1,483 (84.6)	333 (22.5)	18.940	<0.001
	Yes	270 (15.4)	94 (34.8)		
Severity of vision impairment	Low vision	774 (44.2)	151 (19.5)	17.688	<0.001
	Blindness	979 (55.8)	276 (28.2)		
Age at onset of the eye diseases	At birth	187 (10.7)	32 (17.1)	9.985	0.007
	Childhood	581 (33.1)	163 (28.1)		
	Adulthood	985 (56.2)	232 (23.6)		
Impaired ability in performing daily activities	No	868 (49.5)	139 (16.0)	64.974	<0.001
	Yes	885 (50.5)	288 (32.5)		

* *Because only 13 persons rated their family financial status as “good”, “good” and “fair” categories were combined together*.

** *Continuous variables were dichotomized at their median values*.

### Prevalence of MDD, Perceived Need for Mental Healthcare, and Utilization of Mental Health Services

A total of 427 respondents were diagnosed with MDD, of whom 111 perceived a need for mental healthcare but only five had received treatment from mental health specialists. The one-month prevalence of MDD was 24.4%. Rates of perceived need for mental healthcare and use of mental health services among respondents with MDD were 26.0 and 1.2%, respectively.

### Factors Associated With MDD

Results of Chi-square test show that significantly higher prevalence rates of MDD were observed in participants who were males, were 36–55 years and 56+ years old, had an educational attainment of primary school, were never-married and separated, divorced and widowed, were unemployed, rated their family economic status as “poor”, resided in rural areas, had a low level of objective social support, had a low level of utilization of social support, were currently smoking, were currently drinking, were blind, had eye conditions during childhood, and had difficulties in performing daily activities (*P* ≤ 0.029), compared with their corresponding counterparts (Table 1).

In multiple logistic regression, factors significantly associated with MDD were middle age (OR = 1.96, *P* < 0.001), older adulthood (OR = 1.79, *P* = 0.004), being never-married (OR = 1.96, *P* < 0.001), being separated, divorced and widowed (OR = 12.30, *P* < 0.001), a low level of objective social support (OR = 1.83, *P* < 0.001), currently drinking (OR = 1.81, *P* < 0.001), having eye conditions during childhood (OR = 1.89, *P* = 0.005), and having difficulties in performing daily activities (OR = 2.78, *P* < 0.001) (Table 2).

**Table 2:** Factors significantly associated with major depressive disorder in persons living with vision disability.

**Variable**		**Odds Ratio (95%CI)**	**P**
Age-group (years)	16–35	1	
	36–55	1.96 (1.37,2.81)	<0.001
	56+	1.79 (1.20,2.66)	0.004
Marital status	Married, remarried, and cohabiting	1	
	Never-married	1.96 (1.38,2.79)	<0.001
	Separated, divorced, and widowed	12.30 (3.40,44.44)	<0.001
Objective social support^*^	≥6	1	
	<6	1.83 (1.39,2.40)	<0.001
Currently drinking	No	1	
	Yes	1.81 (1.33,2.45)	<0.001
Age at onset of the eye disease	At birth	1	
	Childhood	1.89 (1.21,2.94)	0.005
Impaired ability in performing daily activities	No	1	
	Yes	2.78 (2.18,3.56)	<0.001

* *Continuous variables were dichotomized at their median values*.

## Discussion

### Main Findings

In the literature, few data have been available on mental disorders among PLwVD. To the best of our knowledge, this is the first large-scale study that examines the epidemiology of MDD in PLwVD and needs for and utilization of mental health services in PLwVD with MDD. The main findings of this study are the 24.4% prevalence of MDD in PLwVD and the 26.0% rate of perceived needs for mental healthcare and 1.2% rate of utilization of mental health services in PLwVD with MDD. In multiple regression analysis, age-groups of 36–55 years and 56+ years (vs. 16–35 years), being never-married and separated, divorced, and widowed (vs. married, remarried, and cohabiting), a low level of objective social support, currently drinking, having eye conditions during childhood (vs. at birth), and having difficulties in performing daily activities were significantly associated with MDD in PLwVD.

Compared to surveys using DSM-IV diagnostic criteria and the same timeframe, the 24.4% one-month prevalence of MDD in Chinese PLwVD is 12 times as high as that of the Chinese general population (2.1%) (42), 18 times as high as that of Chinese rural-to-urban migrant workers (1.4%) (43), four times as high as that of Chinese inpatients of general hospitals (6.9%) (44), and twice as high as that of Chinese inpatients with cancers (12.6%) (45). We speculate that the interactions between biological, psychological, and social factors may explain the very high prevalence of MDD in PLwVD. First, there is evidence that the suppression of melatonin secretion increases the risk of depression via its negative impact on sleep-wake rhythm, inhibitory effects on the secretion of monoamine neurotransmitters such as 5-HT and NE, and promoting effects on the secretion of inflammation-related cytokines such as TNF-α and IL-6 (46). Because of the circadian desynchrony induced by lack of intact or no perception of light (47), we speculate that the suppression of melatonin may further heighten the risk of depression in PLwVD. Second, vision loss, as a major stressful life event, can result in a variety of difficulties in everyday life such as recognizing faces, reading, or mobility (48). Since the vision impairments in PLwVD have been irreversible and often progressive, this population is at particularly high risk of experiencing continuous physical and mental sufferings including depression (49). Third, given the substantial evidence that higher risk of depression is associated with a lower socioeconomic status (50), the high prevalence of MDD in our sample may also be attributed to the socioeconomically disadvantaged status of PLwVD; as shown in Table 1, 56.3% of the PLwVD were unemployed, 81.2% had poor family economic status, and 50.5% relied on others to perform daily routines.

Suffering from mental disorders is a major driver of perceiving needs for mental healthcare and for the use of mental health services (51). Given the high prevalence of MDD, PLwVD should have greater needs for mental healthcare. However, compared to the 39.3% rate of perceived needs for mental healthcare in Chinese adults with MDD (52), the 26.0% rate of perceived needs suggests that PLwVD are less likely to seek help from mental health specialists when they are suffering from MDD. We speculate that the poorer mental health literacy and greater intention to self-manage emotional problems in PLwVD might be the potential reasons for the large gap between suffering from MDD and subjective unawareness of the need for mental healthcare.

Depression is often overlooked and untreated in eye care settings; for example, in Great Britain, 25.2% of the patients who were receiving low-vision services and screened positive for significant depressive symptoms were being treated for their depression (53). Nevertheless, the 1.2% rate of mental health services utilization in Chinese PLwVD with MDD is extremely low, suggesting almost none of these persons receive treatment. In China, the underutilization of mental health services is a common phenomenon in various populations with MDD. For example, only 4.7% of the Chinese residents with 12-month MDD were treated by mental health specialists, only 1.5% of the Chinese persons with speech disability with one-month MDD used mental health services, and only 3.3% of the Chinese rural-to-urban migrant workers with lifetime MDD sought treatment from mental health specialists (24, 43, 54). The most commonly reported barriers to mental health treatment in China include inadequate provision of mental health service resources, high cost of mental health services, lack of community-based mental health facilities, negative attitudes toward people with mental disorders and mental health institutions, and stigma surrounding mental illness (42, 52). In the case of PLwVD in China, economic difficulties, lack of accessible public transportation, inadequate mental health training of ophthalmologists, and no PLwVD-friendly mental health services may further prevent them from seeking needed treatment.

Our findings on associations of MDD with age-group, marital status, social support, and ability of activities of daily living in PLwVD are similar to those reported in prior studies on depression in the general population, which identify middle and old age-groups, marital status of “never-married, separated, widowed, or divorced”, alcohol consumption, inadequate social support, and poor ability of activities of daily living as primary risk factors for depression (42, 54–59). Childhood is a critical period of an individual's psychological and behavioral development, making a child sensitive to certain environmental factors (60). Accordingly, exposure to vision loss during this vulnerable period would have profound negative effects on later mental health in humans. Therefore, the greater risk of MDD in PLwVD with childhood-onset eye condtions is expected in this study.

## Limitations

The present study has some limitations. First, data of this study were collected cross-sectionally, so prospective studies are warranted to further confirm whether there are causal relationships between identified factors and MDD. Second, qualitative data on barriers to the use of mental health services and reasons for unawareness of the need for mental healthcare are necessary for effective mental health policy-making in PLwVD, but we did not collect such data. Third, the CDPF registration system did not cover all PLwVD because the registration is not compulsory. Therefore, strictly speaking, our findings can only be generalized to PLwVD who were registered with the CDPF. Nonetheless, the CDPF system provides an efficient network to approach this vulnerable population, who can become an important target group for any subsequent mental health services. Fourth, the comparison of the severity of depressive symptoms between PLwVD with MDD who perceived needs for mental healthcare and those who did not would facilitate the understanding the discrepancy between “clinical need” and “perceived need” for mental healthcare, but we did not assess the severity of depressive symptoms of PLwVD with MDD. Fifth, our assessment on the clinical characteristics of PLwVD is not detailed enough, i.e., no data of types and durations of eye conditions, so more studies are warranted to determine associations of MDD with ophthalmological characteristics.

## Conclusion and Implications

In summary, nearly one-fourth of the Chinese PLwVD have MDD, but only 26.0% and 1.2% of the PLwVD with MDD perceive a need for mental healthcare and seek treatment from mental health specialists, respectively. The high prevalence of MDD and low rates of perceived needs for mental healthcare and utilization of mental health services suggest a high level of mental healthcare needs that are not being met. Given the limited mental health services resources in China, efforts are needed to identify and remove barriers to mental health services in PLwVD. Public strategies such as the integration of mental healthcare into eye care and rehabilitation services and the provision of PLwVD-friendly mental health services are also warranted to make the mental health services available, accessible, and affordable for PLwVD. In addition, mental health education and promotion are also necessary to increase the awareness of the need for mental healthcare.

Mental health services for Chinese PLwVD need to include periodic screening for MDD (and other mental disorders), expanded psychosocial supports, and, when necessary, psychiatric assessment and treatment. Further, services for PLwVD would be more effective if they targeted those who are 36+ years, are never-married, separated, divorced and widowed, have inadequate social support, are current drinkers, have childhood-onset eye conditions, and rely on others to perform activity of daily living.

## Data Availability Statement

The raw data supporting the conclusions of this article will be made available by the authors, without undue reservation.

## Ethics Statement

The studies involving human participants were reviewed and approved by Institutional Review Board of Wuhan Mental Health Center. The patients/participants provided their written informed consent to participate in this study.

## Author Contributions

B-LZ: acquisition and analysis of data for the study, drafting the paper, and interpretation of data for the study. Y-MX: design and acquisition of data for the study. B-LZ and YL: drafting the paper, revising the paper for important intellectual content, and interpretation of data for the study. All authors contributed to the article and approved the submitted version.

## Funding

This work was supported by National Natural Science Foundation of China (Grant Number: 71774060). The funding source listed had no role in study design, collection, analysis and interpretation of data, writing of the report, and in the decision to submit the paper for publication.

## Conflict of Interest

The authors declare that the research was conducted in the absence of any commercial or financial relationships that could be construed as a potential conflict of interest.

## Publisher's Note

All claims expressed in this article are solely those of the authors and do not necessarily represent those of their affiliated organizations, or those of the publisher, the editors and the reviewers. Any product that may be evaluated in this article, or claim that may be made by its manufacturer, is not guaranteed or endorsed by the publisher.
